# Targeting ferroptosis for neuroprotection: potential therapeutic avenues in neurodegenerative and neuropsychiatric diseases

**DOI:** 10.3389/fphys.2025.1641323

**Published:** 2025-08-28

**Authors:** Muhammad S. Khan, Qichan Hu, Kendrix Okeibunor, Liang Ma, Jean C. Bopassa

**Affiliations:** ^1^ Department of Cellular and Integrative Physiology, University of Texas Health Science Center at San Antonio, San Antonio, TX, United States; ^2^ Glenn Biggs Institute for Alzheimer’s & Neurodegenerative Diseases, University of Texas Health Science Center at San Antonio, San Antonio, TX, United States; ^3^ Department of Pharmacology, University of Texas Health Science Center at San Antonio, San Antonio, TX, United States

**Keywords:** ferroptosis, lipid peroxidation, oxidative stress, neurodegeneration with brain iron accumulation (NBIA), aceruloplasminemia, alzheimer’s disease, parkinson’s disease

## Abstract

Ferroptosis is an iron-dependent programmed cell death that plays an important role in neurodegenerative and neuropsychiatric diseases. In the present study, we have highlighted how different risk factors are involved in the induction of ferroptosis in brain cells. In addition, we also demonstrated how ferroptosis plays an important role in different brain diseases. In our study why we focused and elaborated on the mechanisms of ferroptosis only in brain cells (Neurons, oligodendrocytes, and microglia) because they are particularly vulnerable to such kind of cell death. Additionally, brain cells are more dependent on mitochondrial function, iron regulation, and high levels of polyunsaturated fatty acids (PUFAs) as compared to peripheral body cells. Highlighting ferroptosis is more important because it has demonstrated several important mechanisms of neuronal injury and dysfunction which provides a deep understanding of the etiology of various brain diseases that were not sufficiently described by other programmed cell death pathways. Therefore, it has led to the exploration of new therapeutic strategies against various brain diseases and thus targeting ferroptosis-related proteins opens a new therapeutic window for several incurable brain diseases, and various ferroptosis regulators are now under clinical trials. However, their validation as a preclinical therapeutic agent is needed. Interestingly, here in our study we also summarize the most recent potential therapeutic targets and promising interventions which will provide a beam of light for future therapies against major brain diseases.

## Introduction

Ferroptosis is an iron-dependent programmed cell death that has bridging metabolic dysfunction with redox biology. Iron is pivotal in normal physiological conditions such as DNA synthesis, cell division, neurotransmission, cellular respiration, oxygen transport, and cellular metabolism. Ferroptosis is characterized by unique biochemical and morphological changes that distinguish it from other program cells death. The most important features of ferroptosis are alteration in redox balance, iron homeostasis and lipid metabolism. Transferrin, Metal Transporter, and Iron Response Element Binding Protein 2, are important regulators of ferroptosis that induces cell death and modulating intracellular and systemic iron homeostasis. Irons overload initiates oxidation of the acyl tail of unsaturated fatty acid via Fenton reactions which in turn leads to increase the formation of reactive oxygen species (ROS) and lipid peroxidation (Stockwell, 2022; Dix et al., 2024; Jiang et al., 2021; Li et al., 2020; Berndt et al., 2024). Therefore, higher the level of unsaturated fatty acid more will be the ROS production and *vice versa*. Primarily there are two antioxidant systems know as Glutathione (GSH)/Glutathione peroxidase 4 (GPX4) systems and the Coenzyme Q10 (CoQ10)/Ferroptosis Suppressor Protein 1 (FSP1) system which catalyzes the reduction of lipid peroxides. Change in the expression and activity of these molecules is very crucial to understand the fate of a cell. Ten years ago, ferroptosis was first documented as a program cell death of the body which is involved in variety of biological processes, including muscle atrophy, neuron loss, tumor growth, ischemia reperfusion and immune escape. It has been shown that ferroptosis play essential role in health maintaince and in the development of multiple human diseses (Supplementary Figure S1).

**FIGURE 1:**
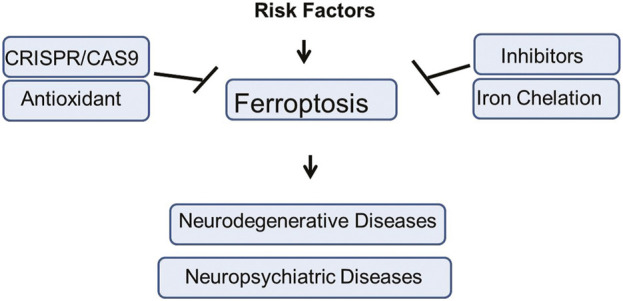
The graphical summery of the present study. The risk factors involved in the induction of Ferroptosis mediated neuropsychiatric and neurodegenerative disorders. Different therapeutic strategies against Ferroptosis mediated brain diseases such as use of inhibitors, antioxidant agents, genetic engineering approach, Iron Chelation, etc., has been elucidated in the present study.

Unlike other forms of cell death, ferroptosis specifically affects neurons with high metabolic demands and polyunsaturated fatty acid-rich membranes, making them especially vulnerable under conditions of oxidative stress and inflammation. Recently, increase interest and development of ferroptosis related research have uncover numeriuos regulators to facilitate the clinical and precilical application of that agents and opend a new therapeutic window for feroptisis related neurodegenerative and psychiatric diseases (Zeng et al., 2023a; Pan et al., 2023; Liang et al., 2022; Tang et al., 2021; Li et al., 2023a; Dixon et al., 2012; Peng et al., 2022a; Liao et al., 2022; Qin et al., 2022). This review comprehensively elaborates the detail mechanisms, importance in brain diseases, regulation, therapeutic avenues and different risk factors of ferroptosis (Figure 1).

We also highlight how to use CRISPR editing on brain disease risk genes in ferroptosis. Our finding could provide strategies for innovative treatments of ferroptosis-associated diseases, offering hope for addressing some of the most challenging biomedical conditions of our time.

## Role and importance of ferroptosis in neurodegenerative, neuropsychatric diseases and its unifying model

**FIGURE 2:**
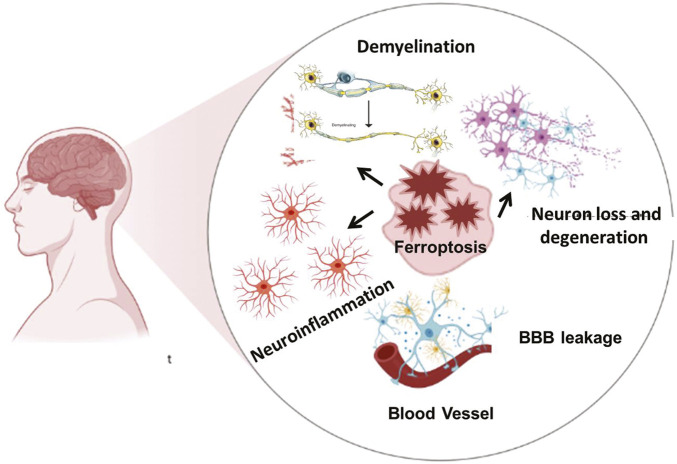
The Ferroptosis mediated brain diseases. Iron overload, ROS burden and oxidative stress are involved in demyelination, neuroinflammation, and neuronal loss which in turn lead to various neurodegenerative and neuropsychiatric diseases. Neuroinflammation is further actively participating in the blood brain barrier disruption.

Iron absorption and transport can be disrupted by neurological disorders, which can result in excessive iron accumulation, elevated oxidative stress, and cellular ferroptosis. Ferroptosis is a major contribution to neurodegenerative disorders, according to growing study (Figure 2) (Wei et al., 2024; Long et al., 2023; Wang et al., 2024). In diseases such as alzheimer’s, parkinson’s, and multiple sclerosis, imbalances in iron metabolism and antioxidant protection result in oxidative stress, rendering neurons and glial cells particularly vulnerable to ferroptotic injury. Microglia and oligodendrocytes, responsible for regulating neuroinflammation and myelination, can experience ferroptosis due to chronic inflammation and oxidative stress, exacerbating neural injury (Li et al., 2024a). In diseases such as alzheimer’s, parkinson’s, and multiple sclerosis, imbalances in iron metabolism and antioxidant protection result in oxidative stress, rendering neurons and glial cells particularly vulnerable to ferroptotic injury. Microglia and oligodendrocytes, responsible for regulating neuroinflammation and myelination, can experience ferroptosis due to chronic inflammation and oxidative stress, exacerbating neural injury (Li et al., 2024a). We have described the ferroptosis drivers across diseases in the following table.

## Unifying model of ferroptosis drivers across diseases

**Table:** 

Driver	Mechanism	Impact in disease context
Iron Accumulation	Excessive iron increases Fenton reactions, generating hydroxyl radicals that promote lipid peroxidation	- AD: Iron overload in cortex/hippocampus - PD: Accumulation in substantia nigra - Depression: Dysregulated systemic iron
Lipid Peroxidation (PUFA-PLs)	Polyunsaturated fatty acids (PUFAs) in membranes are oxidized under stress, forming toxic lipid ROS like 4-HNE and MDA.	- AD: Elevated 4-HNE in neurons - PD: Lipid oxidation damages dopaminergic neurons - Depression: Associated with inflammation
GPX4 Inhibition	Glutathione peroxidase 4 reduces lipid peroxides; its depletion or inactivation leads to ferroptosis	- All: GPX4 downregulated in affected brain regions
Glutathione (GSH) Depletion	GSH is the primary cofactor for GPX4; low levels impair antioxidant defenses	- AD/PD: Low GSH in aging brain - Depression: Reduced GSH in plasma and brain
Increased ACSL4 Expression	ACSL4 integrates oxidizable PUFAs into membranes, sensitizing cells to ferroptosis	- PD: Increases ferroptosis in dopaminergic neurons - Depression/AD: ACSL4 underexplored, emerging evidence
NRF2 Pathway Dysfunction	NRF2 regulates antioxidant defenses including GPX4 and ferritin. Its suppression exacerbates ferroptosis	- AD: Impaired nuclear translocation - PD: Suppressed in SN - Depression: Blunted antioxidant gene expression
Inflammatory Cytokines	IL-1β, TNF-α, and IL-6 induce iron uptake, inhibit antioxidant enzymes, and enhance ROS production	- All: Inflammatory milieu drives iron dysregulation and ferroptosis
Mitochondrial Dysfunction	Mitochondria generate ROS and regulate iron metabolism; dysfunction leads to ferroptosis sensitivity	- AD/PD: Common feature - Depression: Linked to metabolic stress and ROS
Dysregulated Ferritin/FPN1	Loss of iron export (via FPN1) or ferritin degradation increases labile iron pool	- AD: Reduced FPN1 in cortex - PD: Impaired ferritin function - Depression: May contribute to iron dyshomeostasis
Excitotoxicity/Glutamate	Excess glutamate inhibits cystine uptake (xCT), reducing GSH and promoting ferroptosis	- AD: Excitotoxic damage - PD: Glutamate overactivity - Depression: Glutamatergic dysfunction contributes to stress response

## Ferroptosis and alzheimer

Abnormal iron metabolism has been linked to the development of AD, according to a number of research. Ferroptosis-related characteristics, such as aberrant iron metabolism, glutamate excitotoxicity, and the buildup of lipid ROS, have been seen in the brain tissues of AD patients and AD model mice. Iron levels in the hippocampus, cortical lobe, and basal ganglia are also higher in AD patients than in control participants, according to research. The degree of amyloid deposition has also been linked to the levels of iron and ferritin in brain tissue. Furthermore, AD patients exhibit downregulated expression of GPX4 and increased levels of 4-HNE and Malondialdehyde in different parts of the brain. According to one study, patients with AD were able to live better lives after receiving an intramuscular injection of the iron-chelating drug deferoxami (Bulk et al., 2018; Lee and Lee, 2019; Yan and Zhang, 2019; Dare et al., 2020; Villalon-Garcia et al., 2023; Rogers and Lahiri, 2004; Lei et al., 2019; Zhang et al., 2022a). One known contributing element to the onset of AD is glutamate excitotoxicity. According to Zhang et al. (2020), AD may result from an increase in extracellular glutamate concentration brought on by system Xc–failure during ferroptosis. Additionally, Feng et al. (2023) found that the hippocampus of a mouse model of AD in which PSEN1 (Presenilin-1) had been knocked down had higher expression of ferroptosis-related proteins (GPX4, SLC7A11, ACSL4, Phosphatidylethanolamine Binding Protein 1) than did healthy mice (Feng et al., 2024a). Recent studies emphasize the importance of iron in controlling tau phosphorylation and aggregation, which may lead to the development of neurofibrillary tangles in neurodegenerative disorders like alzheimer’s disease. A notable interaction seems to exist between iron and tau, affecting disease progression and symptoms. Notably, tau binds to iron, resulting in its aggregation and potential accumulation as iron-rich tangles in the brains of individuals with AD. Additionally, increased iron concentrations may enhance tau phosphorylation in cultured neurons, indicating a possible connection between increased iron and abnormal tau in alzheimer’s disease (Mohammadi et al., 2024). Furthermore, Recent studies suggest that the Iron-driven production of reactive oxygen species (ROS) could result in protein misfolding and cell damage. The misfolding and aggregation of neuronal proteins like α-synuclein, Tau, amyloid beta (Aβ), TDP-43, or SOD1 is a prevalent characteristic of various neurodegenerative diseases, and iron has been demonstrated to promote protein aggregation (Joppe et al., 2019). This suggests that ferroptosis and neurodegenerative diseases like AD are closely related. Because of this, ferroptosis could be a key process in the development of AD. Ferroptosis, driven by iron accumulation, oxidative stress, and protein aggregation, plays a central role in the pathogenesis and progression of Alzheimer’s disease (Dare et al., 2020; Feng et al., 2024a; Zhang et al., 2020; Han et al., 2023).

## Ferroptosis and parkinson

Ferroptosis and Parkinson’s disease are closely related, according to an increasing number of studies. Ferroptosis-like clinical features of parkinson’s disease (PD) include oxidative stress, LPO, GSH depletion, and abnormalities of iron metabolism (da Costa Caiado et al., 2025). Furthermore, the loss of dopaminergic neurones in the substantia nigra (SN) and striatum can be avoided by using the iron chelating drug DFP and the ferroptosis-specific inhibitor Ferrostatin-1 (Fer-1) Lipostatin-1. Ferroptosis was identified by Do Van et al. (2016) as a new type of cellular death in parkinson’s disease. In dopaminergic cells, Erastin causes cytotoxicity through activating Protein Kinase C (PKC), which sets off MEK signalling and promotes ferroptosis. Additionally, ferroptosis in PD can be reduced by PKC suppression. The 140 amino acid protein known as α-Syn is mostly expressed in the brain and is essential for several neuronal synaptic functions. One important pathological sign of parkinson’s disease (PD) is the aggregation of α-Syn, which is a prominent component of intracellular Lewy bodies. α-Syn produces LPO by producing ROS, which increases calcium influx and causes cell death (Dionisio et al., 2021; Thapa et al., 2022; Do Van et al., 2016; Chartier-Harlin et al., 2004; Angelova et al., 2020). Cumulative findings showed that lowering α-synuclein levels in dopaminergic neurons helps prevent ferroptosis, whereas enhanced α-synuclein levels in neuronal precursor cells from patients with SNCA triplication increases susceptibility to lipid peroxidation and ferroptosis (Mahoney-Sanchez et al., 2022). Ferroptosis has a role in the degenerative mechanism of parkinson’s disease (PD), as research has shown that elevated iron accumulation or decreased intracellular glutathione levels contribute to the abnormal aggregation of PD α-Syn (Angelova et al., 2020). Ferroptosis contributes significantly to Parkinson’s disease progression through iron accumulation, oxidative stress, and α-synuclein aggregation, making it a promising therapeutic target.

## Ferroptosis and depression

According to research, neuroinflammation is the immune response of the central nervous system (CNS) that is mostly the result of astrocytes and microglia in the hippocampus. A possible link between iron and neuroinflammation has been suggested by the association of microglia, which are recognised for having a high iron content, with depression linked to aberrant glial activation and iron overload. It is unknown, therefore, exactly how iron overload upsets neurotransmitter balance and causes anxiety and depressed symptoms (Li et al., 2024a; Lee and Hyun, 2023; Uzungil et al., 2022; Zeng et al., 2023b). Recent studies shown that In depression and associated neuropsychiatric conditions, inflammatory mechanisms are crucial in promoting ferroptosis. Increased concentrations of pro-inflammatory cytokines—like TNF-α, IL-6, and IL-1β—can interfere with glutathione metabolism and inhibit antioxidant systems such as GPX4, making neurons more susceptible to oxidative damage. At the same time, inflammation causes iron imbalances, raising intracellular free iron by enhancing DMT1 expression and degrading Ferritin, which further promotes lipid peroxidation. These mechanisms establish a feedback loop in which persistent neuroinflammation drives ferroptosis, leading to neuronal degeneration and the underlying causes of depression (Liu et al., 2025; Feng et al., 2025; Dang et al., 2022).

Studies have demonstrated that Brain-derived Neurotrophic Factor (BDNF) signal transduction is essential for synaptic plasticity in depression, and that BDNF downregulation may have neurotoxic consequences. According to Li et al., iron overload may cause BDNF to be downregulated through the iron urin BDNF pathway, which could result in injury to the hippocampus. Additionally, Gao et al. demonstrated that iron deposition in hippocampal microglia is directly linked to neuronal death and degeneration in a Chronic Unexpected Mild Stress (CUMS) animal model. Furthermore, Zeng et al. emphasised how important Nrf2 is as an anti-inflammatory mediator in controlling iron deposition and neuroinflammatory reactions in depression. Cao et al. found clear changes in protein expression between normal mice and CUMS model animals in a comparative research employing hippocampus proteomics, showing significant iron deposition and neuronal necrosis activation in the hippocampus, which encourages the development of depression. Zhang et al. recently found that CUMS model mice had considerably higher expression levels of different inflammatory markers, but that this neuropathological alteration was successfully reversed by treatment with the iron chelating drug deferoxamine (DFO). All of these results point to a possible connection between the onset of depression and the neurotoxicity brought on by iron overload (Zeng et al., 2023b; Shkundin and Halaris, 2023; Li et al., 2023b; Gao et al., 2019; Cao et al., 2021; Zhang et al., 2022b; Lima Giacobbo et al., 2019; Williams et al., 2022). Iron overload–induced neuroinflammation and ferroptosis play a central role in the pathogenesis of depression by disrupting antioxidant defenses, impairing BDNF signaling, and triggering neuronal degeneration. In order to elaborate the ferroptosis related marker and their correlation with neurodegenerative and neuropschychtric diseases, we have provided a summarized table.

**Table:** 

Marker	Alzheimer’s disease (AD)	Parkinson’s disease (PD)	Depression	Key references
GPX4	↓ Expression in hippocampus and cortex	↓ Levels in substantia nigra	↓ Activity observed in animal models; may underlie oxidative damage	Zhang et al. (2024a)
GSH	↓ Brain levels in AD patients	↓ Levels in SN; associated with dopaminergic neuron loss	↓ Serum levels in MDD; linked to treatment-resistant depression	Mazzetti et al. (2015), Gu et al. (2015)
4-HNE	↑ Accumulated in AD brain tissues	↑ Found in dopaminergic neurons in PD	↑ Linked to inflammation and oxidative damage in depressive brains	Zarkovic (2003); Mattson (2009)
ACSL4	↑ Expression in neurons prone to ferroptosis	↑ Associated with neuronal susceptibility to oxidative damage	Potential ↑ in stress-induced models; underexplored in depression	Chen et al. (2023a), Zhuo et al. (2025)
TFRC (TfR1)	↑ Expression in AD neurons and microglia	↑ Found in substantia nigra; enhances iron influx	↑ Expression linked to inflammatory response and oxidative stress	Petralla et al. (2024), Helgudottir et al. (2024)
FPN1	↓ Expression in cortex and hippocampus	↓ Causes intracellular iron accumulation in SN	↓ May enhance cellular iron retention under inflammatory states	Qian et al. (2023), Gao et al. (2023)
HO-1	↑ Overexpressed in astrocytes; may contribute to redox imbalance	↑ Observed in PD patients; associated with glial activation	↑ Induced by inflammation and chronic stress in rodent depression models	Neis et al. (2018), Sebghatollahi et al. (2025)
Lipid ROS	↑ Found in AD mouse models	↑ Detected in midbrain and striatum	↑ ROS production observed in stress models	Uttara et al. (2009), Houldsworth (2024), Olufunmilayo et al. (2023)
NRF2	↓ Activity and nuclear translocation in AD	↓ Impaired in PD; restoration provides neuroprotection	↓ Activity correlates with oxidative damage and mood disorders	Suzen et al. (2022), Brandes and Gray (2020)
FTH1	Altered—↓ in neurons, ↑ in glia	Dysregulated; iron mismanagement observed	↓ Expression can sensitize cells to ferroptosis	Muhoberac and Vidal (2019), Shieh et al. (2023)

## Mechanisms of ferroptosis

Aging and metabolic impairment could affect iron’s normal physiological function in the body, increasing the risks of iron-linked neurodegenerative diseases. Specifically, the free intracellular divalent iron (Fe^2+^) is highly reactive, as it promotes the generation of Reactive Oxygen Species (ROS) via Fenton reactions. These ROS catalyze the peroxidation of polyunsaturated fatty acids, resulting in cellular membrane damage. Aging-dependent iron accumulation in the brain promotes direct ferroptosis and enzyme-mediated redox reactions such as lipid peroxidation (Dixon et al., 2012; Li et al., 2021; Benarroch, 2023; Zhou et al., 2020a; Rochette et al., 2022; Sato et al., 2022; Zhou et al., 2023). Ferroptosis-linked Neurodegenerative Diseases (NDDs) encompass a complex group of conditions associated with neuronal cell death and functional decline (Sun et al., 2022). Advances in proteomic, genomic, animal, and cellular approaches have identified several novel targets recently approved for treating alzheimer’s Disease (AD), Amyotrophic Lateral Sclerosis (ALS), and other NDDs (Emerson and Swarup, 2023; Amartumur et al., 2024). Several convincing studies have revealed that iron accumulation in affected brain regions and multiple neurodegenerative diseases share pathological links. The concept that iron overload significantly accelerates neurodegeneration and acts as a driver of disease progression is increasingly supported by research. (Stockwell, 2022; Dixon et al., 2012; Sato et al., 2022; Ndayisaba et al., 2019; D’Mello and Kindy, 2020; Mitchell et al., 1973; Wiernicki et al., 2020; Kurian and Hayflick, 2013).

In alzheimer’s disease, iron dysregulation contributes to amyloid-beta plaque aggregation and tau hyperphosphorylation. Both processes exacerbate oxidative stress and neuronal damage, ultimately resulting in progressive cognitive decline (Tamagno et al., 2021). Iron deposition in the hippocampus and basal ganglia of AD patients correlates with disease severity and is linked to impaired memory and executive function (Wang et al., 2023a; Liu et al., 2018). Similarly, ALS is characterized by motor neuron degeneration, muscle atrophy, and eventual paralysis (Wijesekera and Leigh, 2009). Iron accumulation in motor neurons has been shown to contribute to mitochondrial dysfunction and oxidative stress, key drivers of disease progression (Cheng et al., 2022). Elevated ferritin levels in the cerebrospinal fluid of ALS patients suggest disrupted iron metabolism as a significant pathological feature (Muyderman and Chen, 2014; Hemerkova and Valis, 2021; Paydarnia et al., 2021; Zheng et al., 2017).

In parkinson’s disease (PD), excess iron in the substantia nigra is associated with dopaminergic neuronal loss and the aggregation of alpha-synuclein, a hallmark of PD pathology. Iron-induced oxidative stress exacerbates neuroinflammation, further promoting neuronal death and motor dysfunction. Therapeutic strategies targeting iron accumulation, such as chelation therapy, show promise in reducing oxidative stress and improving motor symptoms in PD (Yi et al., 2022; Srinivasan et al., 2021).

Neurodegeneration with Brain Iron Accumulation (NBIA) disorders, such as Pantothenate Kinase-Associated Neurodegeneration PKAN, are rare conditions involving abnormal iron deposition in the basal ganglia. Clinically, these disorders present with progressive movement abnormalities, dystonia, parkinsonism, and cognitive decline. The excessive iron in these regions triggers ferroptosis, leading to neuronal degeneration and the observed neurological deficits (Tonekaboni and Mollamohammadi, 2014; Dusek et al., 2022).

Understanding the interplay between ferroptosis and these neurodegenerative diseases is crucial for developing therapeutic strategies aimed at preventing ferroptosis-mediated neuronal damage. By targeting iron dysregulation, oxidative stress, and lipid peroxidation, researchers hope to mitigate the progression of these debilitating conditions.

Recent research indicates lipid and amino acid metabolism provides a foundation for ferroptosis. For example, in 1973, Jerry Mitchell revealed that acetaminophen induces hepatic necrosis in rats dependent on Cysteine and glutathione (GSH). Similarly, the polyunsaturated fatty acids in the membrane lipids were identified as an essential peroxidation substrate for ferroptosis (Stockwell, 2022; Mitchell et al., 1973). However, ferroptosis has multiple molecular regulators, and it is still unclear whether it is programmed cell death because the molecular pathway in normal physiology has not yet been well explored. Multiple pathways need to be explored. However, the most recent and studied mechanisms involved in regulating ferroptosis are loss of the antioxidant system of the cell, iron dyshomeostasis, and lipid peroxidation (Stockwell, 2022; Dixon et al., 2012; Wiernicki et al., 2020).

## Redox reaction imbalance and ferroptosis

**FIGURE 3:**
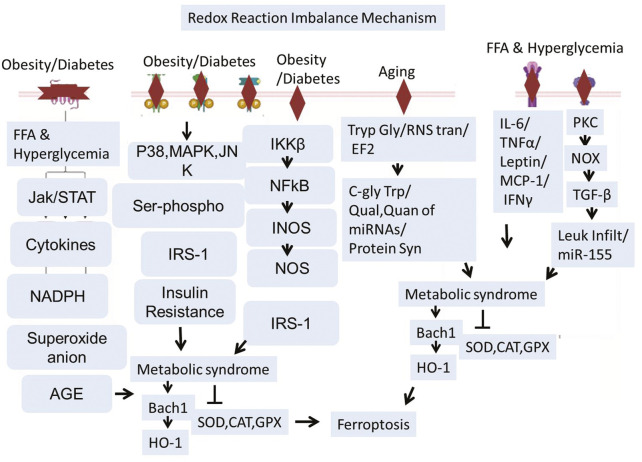
Schematic representation illustrating how aging, Type 2 Diabetes (T2D), and obesity lead to ferroptosis and neurodegeneration. Aging, T2D, and obesity hinder antioxidant defenses by affecting RNA transfer, lowering TEF2 activity, and triggering chronic inflammation. These conditions trigger oxidative stress pathways such as JAK/STAT, NF-κB, and JNK, resulting in decreased activity of essential antioxidant enzymes (SOD, CAT, GPX). Increased free fatty acids, AGEs, and cytokines intensify ROS generation and insulin resistance. Ferroptosis is facilitated by BACH1-driven inhibition of GSH production alongside HO-1–triggered iron accumulation and lipid peroxidation. The consequent ferroptotic cell death aids in neurodegeneration and demyelination via TCR signaling.

Active factors disrupting the antioxidant system-mediated ferroptosis are aging, Type 2 Diabetes (T2D), chronic obesity, etc. (Deng et al., 2023; Li et al., 2023c). Aging could negatively affect the transfer of RNA between cells (Dluzen et al., 2017; Hamdan et al., 2021), factors involved in protein syntheses such as Translation Elongation Factor 2 (TEF2), and the level of C-Glycosyl Tryptophan. Increased glycosylation of tryptophan and increased (C-gly Trp) strongly correlate with aging (Li et al., 2023c; Anisimova et al., 2018; Parrado et al., 1999; Menni et al., 2013; Schmidt-Sommerfeld et al., 1992; Il’yasova et al., 2012; Cindric et al., 2021). Translation Elongation Factor 2 (TEF2) is relatively less active and more fragmented with age, which results in the decline of protein synthesis. This phenomenon induces ROS, and, in turn, this reactive oxygen species inhibits the activation of TEF-2 (Parrado et al., 1999; Li et al., 2024b; Ball et al., 2021). Dysregulated RNA transfer is related to the poor quantity and quality of MicroRNAs (miRNAs). All these aging-related impairments ultimately affect cell growth, cell survival, protein synthesis, and antioxidant defense mechanisms of cells. Recent research revealed that metabolic syndrome and metabolic panel dysregulation are strongly linked with diabetes and obesity (Ball et al., 2021; Iorio and Croce, 2012; Bonomini et al., 2015; Zhang et al., 2023a). T2D and obesity could trigger multiple pathways, such as the inflammatory pathways that include activation and translocation of Necrotic Factor Kappa B (NF-kB), TNFα, INOS, IL-1β, INF-γ, leukocyte infiltration, MCP-1, etc (Mahmoud and Abdel-Rasheed, 2023; Marunaka, 2023; Khan et al., 2021). Increased free fatty acid and hyperglycemia are the two most common worst conditions in obese diabetic patients. This could activate both insulin-resistant and oxidative stress pathways (Khan et al., 2021; Tangvarasittichai, 2015; Fryk et al., 2021; Chandrasekaran and Weiskirchen, 2024). Aberrant activation of the JAK/STAT pathway is linked with the induction of inflammatory cytokines, producing Superoxide Anions (SA) and Advanced Glycation End Products (AGEs) (Simon et al., 1998). Stress Kinase (JNK) and transcription factor Nuclear Factor kappa B (NF-κB) activation and phosphorylation actively induce insulin resistance via Insulin Receptor Substrate 1 (IRS1) disruption (Solinas and Becattini, 2017; Yung and Giacca, 2020; Baker et al., 2011). Collectively, Age, T2D, and obesity-related signaling pathways end at induction of oxidative stress and disruption of first-line defense Antioxidants-Superoxide Dismutase (SOD), Catalase (CAT), and Glutathione Peroxidase (GPX) (Novak et al., 1996; Wang and Zhang, 2024; Zgutka et al., 2023; Promyos et al., 2023; Gusti et al., 2021). On the other hand, it can also regulate BTB and CNC Homology 1 (BACH1) and Heme oxygenase-1 (HO-1) (Kondo et al., 2013; Jin et al., 2023; Ryter, 2022). BACH1 promotes ferroptosis by repressing gene transcription that regulates Glutathione (GSH) synthesis and intracellular labile iron metabolism (Irikura et al., 2023; Soni et al., 2024). At the same time, HO-1 can act as a mediator of ferroptosis. HO-1 can increase the labile iron pool and promote lipid peroxidation, leading to ferroptosis (Han et al., 2022; Chen et al., 2023b). Ferroptosis in the brain works very closely with the demyelination of the neuronal cells, which is dependent on T Cell Receptor (TCR) signaling (Luoqian et al., 2022; Qin et al., 2023). Aging, Type 2 Diabetes, and obesity converge on oxidative stress and inflammatory pathways that disrupt antioxidant defenses and promote ferroptosis, contributing to neurodegeneration and demyelination (Figure 3).

## Abnormal iron metabolism and ferroptosis

There are several well known health condition which aguments iron dyshomeostasis and activates different pathways that leads to iron load and lipids peroxidation in the central nervous system. Among them, Cardiovascular diseases, high cholesterol, smoking, diabetes, and high blood pressure are the common abnormilities that could induces cerebral ischemia and stroke. These factors lead to the building of plaques and clots in the arteries, which lead to a lack of blood flow to the brain (Ekker et al., 2023). The other causes like many babies develop hypoxic conditions in their brains if the oxygen is not distributed correctly to the brain immediately after birth. This sometimes creates a group of conditions collectively known as cerebral palsy (CP) (Paul et al., 2022). Therefore, the proper distribution of oxygen in the body is necessary. Poor, insufficient, or lack of oxygen supply to the organ results in hypoxia, infarction, or ischemia, which aggravates several cell death pathways mediated through lipid peroxidation and iron overload. Cerebral infarction and brain ischemia mediate Ubiquitin-Specific Protease 14 (USP14), Cyclic Guanosine Monophosphate–Adenosine Monophosphate Synthase cGAS-STING, JAK-STAT3, HIF-1α, and Nrf-2 signaling (Zhang et al., 2023b; Ma et al., 2023; Huang et al., 2022; Li et al., 2023d; Liu et al., 2024a; Hu et al., 2022). Recent studies showed that USP14 activation is involved in iron overload, while inhibition enhances mitophagy and normalizes the mitochondrial defects of Parkin KO human neurons (Bernardo et al., 2024). Hypoxic damage in the brain could also activate HIF-1α, which is a transcription complex. HIF-1α can increase iron levels in the brain via upregulating Transferrin Receptor 1 (TfR1) (Baranova et al., 2007; Vela, 2018). TfR1 is involved in transporting iron in the cell (Vela, 2018; Ding et al., 2011; Wang et al., 2020a; Fillebeen et al., 2019). Besides cerebral ischemia, Traumatic Brain Injury (TBI) is a leading cause of brain damage and paralysis. Kids and Athletic people are more vulnerable to such kinds of injuries. TBI not only causes acute brain damage but also sometimes leads to chronic and long-term disabilities. Recently, several studies have explored the mechanisms involved in TBI-related brain pathologies. Besides other pathologies, TBI is also actively involved in brain iron dyshomeostasis and overload via inhibition of the TrkB/PI3K/Akt/Nrf2 signaling pathway. Other conditions like metabolic disorders could induce iron overload via endoplasmic reticulum stress, ROS, and suppression of cytokine signaling three expressions. A recent study highlighted the critical role of hypothalamic iron in obesity development. This study revealed that reducing iron overload in AgRP neurons inhibits AgRP neuron activity, endoplasmic reticulum stress, Suppressor of Cytokine Signaling 3 (SOCS3), oxidative stress, and NF-κB signaling. This mechanism works like a feedback loop where iron overload induces obesity, and on the other hand, obesity and metabolic disorders will accelerate iron overload (Zhang et al., 2024b; Li et al., 2023e). The aging process could connected with increased hepcidin, while increased hepcidin is associated with increased ubiquitination. This could significantly reduce the iron exporters known as Ferroportin-1 (FPN1) (Sato et al., 2022). Various health conditions—such as cardiovascular disease, cerebral ischemia, hypoxia, traumatic brain injury, metabolic disorders, and aging—converge on iron dyshomeostasis and oxidative stress pathways, promoting ferroptosis and contributing to neurodegeneration.

**FIGURE 4:**
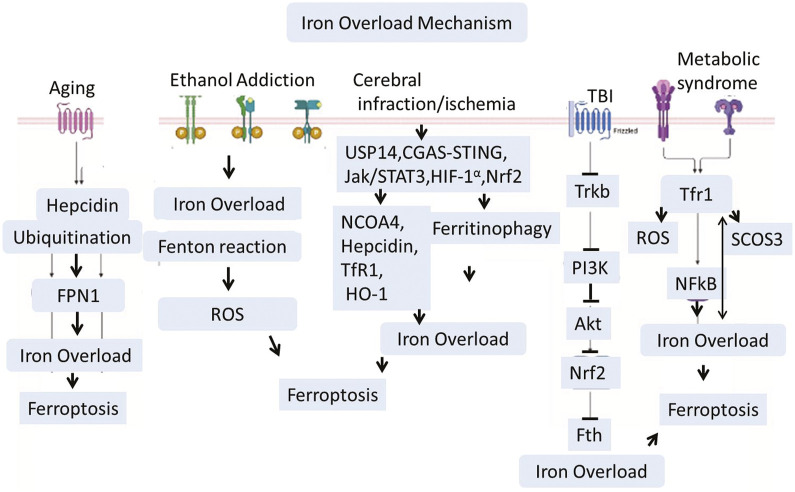
Traumatic brain injury (TBI), Ischemia, Aging, and ethanol addiction interfere with iron metabolism via signaling pathways including USP14, HIF-1α, and JAK/STAT3. Iron accumulation is worsened by heightened expression of Transferrin Receptor 1 (TfR1) and decreased iron export through Ferroportin-1 (FPN1), especially in older individuals. Furthermore, the buildup of iron in the hypothalamus linked to obesity encourages oxidative stress and inflammation via SOCS3 and NF-κB. Together, these mechanisms promote lipid peroxidation and ferroptosis, connecting systemic issues to neurodegeneration in conditions like Alzheimer’s through tau hyperphosphorylation and amyloid dysregulation.

Iron overload has been implicated in the development of neurodegenerative diseases such as alzheimer’s via increased tau phosphorylation and abnormal cleavage of amyloid precursor proteins, as shown in Wang et al. (2023a), Wang et al. (2022), Wang et al. (2020b) (Figure 4).

## Lipid metabolism and ferroptosis

**FIGURE 5:**
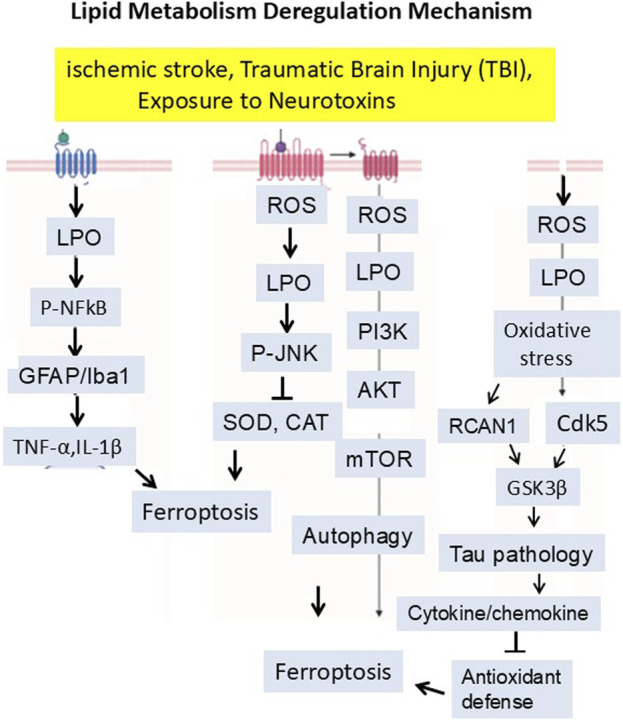
Mitochondria-derived reactive oxygen species (ROS) initiate lipid peroxidation, which disrupts membrane integrity and activates signaling pathways—such as PI3K/AKT/mTOR inhibition, CDK5–GSK3β–Tau hyperphosphorylation, and JNK–NF-κB-mediated neuroinflammation—ultimately promoting ferroptosis and neurodegeneration. The signaling mechanism has been summarized in the manuscript.

Many types of brain injury, insult, or stress could induce the production of Reactive Oxygen Species (ROS). During the oxidative phosphorylation process within the mitochondria, many electrons leak from the electron transport chain, interacting with the oxygen molecule and producing superoxide radicals (O2-). They are byproducts of normal metabolism in the body. These superoxide radicals are converted into other ROS like hydrogen peroxide (H_2_O_2_) and Hydroxyl Radicals (HO-). When the cellular antioxidant system cannot control and balance this reactive species, it will generate oxidative stress (Pizzino et al., 2017; Brieg et al., 2012). These free radicals are very reactive and attack unsaturated fatty acids in a cell membrane, known as lipid peroxidation. Lipid peroxidation is a chain reaction that damages the cell membranes (Pre, 1991). Lipid peroxidation causes ferroptosis by activating or inhibiting several signaling pathways. Recent studies have shown that LPO inhibits the PI3K/AKT/mTOR signaling pathway. Inhibiting the PI3K/AKT/mTOR pathway can increase autophagy. Excessive autophagy can lead to iron accumulation and higher oxidative stress levels, amplifying ferroptosis (Butler et al., 2017; Yang et al., 2023; Zhang et al., 2023c). It is essential to know that LPO-mediated oxidative stress burden can trigger the expression of Regulator of Calcineurin 1 (RCAN1) (originally called Adapt78) and Cyclin-Dependent Kinase 5 (CDK5). In old animals, it has been observed that CDK5 over-activation significantly triggers the GSK3 beta activities, which in turn leads to Tau hyperphosphorylation (Ermak et al., 2011; Guo et al., 2018; Engmann and Giese, 2009; Plattner et al., 2006; Lloret et al., 2011). It is well-recognized that lipid peroxidation also activates other stress-related pathways that come with ferroptosis. The most relevant is the phosphorylated JNK pathway. JNK activates the transcription factors such as NF-κB and initiates the release of cytokines and chemokines. These cytokines work in both ways, i.e., on one side, they disrupt the antioxidant system, while on the other, they induce microgliosis, astrocytosis, and neuroinflammation. LPO induces ferroptosis, which is involved in amyloid beta aggregation and neurodegeneration (Figure 5) (Banji et al., 2022; Khan et al., 2016).

Besides these commen ferrosptosis mechanisms, there are several well documented studies which indicated that the cellular iron is mainly processed in the cytoplasm, mitochondria, and endosomes, where it experiences uptake, utilization, storage, and regulation. Iron is taken up by the cell through transferrin receptors, released in endosomes where it is reduced from Fe^3+^ to Fe^2+^, and then moved into the cytoplasm by divalent metal transporter 1 (DMT1). In the cytoplasm, surplus iron is securely stored in ferritin, while mitochondria use iron for producing heme and iron-sulfur (Fe-S) clusters, crucial for cellular respiration and enzyme functions. Iron dysregulation and build-up significantly affect mitochondrial performance, as mitochondria are key consumers and controllers of cellular iron. An overload of iron in mitochondria stimulates the production of reactive oxygen species (ROS) via Fenton reactions, resulting in oxidative damage to mitochondrial DNA, proteins, and lipids. This hinders electron transport chain function, lowers ATP synthesis, and interferes with mitochondrial membrane potential. With time, these alterations can induce mitochondrial dysfunction, facilitating cell death mechanisms like ferroptosis. Additionaly, Iron dysregulation and excess significantly affect mitochondrial dynamics, resulting in disrupted movement, fusion/fission balance, and mitophagy. This disturbs the mitochondrial membrane potential and impacts motor proteins crucial for correct mitochondrial transport along axons and dendrites. Moreover, impaired mitochondria do not efficiently undergo mitophagy as a result of oxidative changes to mitophagy receptors and hindered autophagosome development. The buildup of impaired mitochondria leads to energy shortages, neuroinflammation, and the advancement of neurodegeneration, as observed in conditions like parkinson’s and alzheimer’s diseases (Tang et al., 2021; Cheng et al., 2022; Bharat et al., 2023; Ru et al., 2024; Onukwufor et al., 2022; Zhao et al., 2024a; Duan et al., 2022a; Chen and Chan, 2009; Chen et al., 2023c; Zong et al., 2024; Beal, 1995; Wang et al., 2023b; Mishra et al., 2022; Wen et al., 2025; Feng et al., 2023; Fang et al., 2023).

## Methodological approaches

This review article aims to summarize the findings of studies on ferroptosis’s mechanism and therapeutic approach. The motivation for preparing this review was based on our previous studies on ferroptosis. Here, we searched for potential research articles on ferroptosis and its mechanisms. In addition, to identify studies on the mechanism and therapeutic strategies of ferroptosis, we conducted searches using the keywords “ferroptosis,” ferroptosis mechanisms,” and “therapeutic strategies” in all available and independent databases. The abstracts were thoroughly studied, and the main findings were recorded to understand these studies clearly. All studies covering animal and cellular models were included.

## Potential therapeutic strategies

It has been reported that artesunate prevents brain damage at low doses by blocking ferroptosis, and iron chelators such as DFO and DFP have demonstrated positive effects against iron-related neurodegenerative disorders. In tauopathy, Dihydroartemisinin (DHA) may have a neuroprotective effect via interacting with O-GlcNAcylation and phosphorylation, pointing to a possible treatment for tau pathology-related learning and memory impairments (Xia et al., 2021). In mice fed a high-fat diet, grape seed extract lowers calcium and iron levels and acts as an antioxidant to prevent ferroptosis. Iron regulatory proteins are crucial for preserving mitochondrial and cellular iron homeostasis. By blocking the iron regulating protein in dopaminergic neurones, BJP-IVb lowers iron content to stop parkinson’s disease. Additionally, rapamycin lessens the substantia nigra’s dopamine neurone loss via controlling ferroptosis and ferritinophagy. By inhibiting ferroptosis, the iron absorption inhibitor ferristatin II offers neuroprotection, while HBED therapy reduces secondary damage following TBI by attaching to Fe^2+^ and changing it into Fe^3+^. Only DFO, DPF, and DFX are presently authorised for clinical usage. (Xia et al., 2021; Kong et al., 2019; Tsurusaki et al., 2019; Guan et al., 2021; Du et al., 2019; Du et al., 2021; El Ayed et al., 2017; Manolova et al., 2019; Li et al., 2024c; Liu et al., 2023a; Khalaf et al., 2018; Yan et al., 2021; Ge et al., 2021).

Sulfasalazine’s neuroprotective properties can be used therapeutically to prevent catastrophic neuronal death (Ryu et al., 2003). By regulating neuroinflammation and ferroptosis through the Nrf2/HO-1 signalling pathway, astragaloside IV reduces stroke-induced early brain damage (Zhang et al., 2023c). By preventing ferroptosis, curcumin protects against disease by upregulating Nrf2 expression and its downstream targets, HO-1 and GPX4, in hepatocytes, cardiomyocytes, neurones, renal tubule cells, and chondrocytes. Eriodictyol inhibits ferroptosis by stimulating the Nrf2/HO-1 pathway, which greatly improves cognitive impairments. Similar processes are used by forsythiin A, salidroside, tetrahydroxy stilbene glycoside, and spermidine to prevent ferroptosis in AD, PD, and myocardial I/R injury. By triggering the Nrf2 pathway and upregulating the expression of GPX4 and SLC7A11. morroniside prevents ferroptosis in dopaminergic neurones in parkinson’s disease. Through the SIRT1/Nrf2 signalling pathway, edaravone prevents ferroptosis and may be used as a treatment for depression, traumatic brain injury, and stroke. By activating Nrf2, Tertiary butylhydroquinone (TBHQ) and hinokitiol have also been demonstrated to have neuroprotective effects (Zhang et al., 2023c; Pardieu et al., 2022; Luo and Zhang, 2021; Peng et al., 2022b; Wang et al., 2023c; Du et al., 2023; Wang et al., 2021).

By downregulating ACSL4 expression independently of PPAR-γ, rosiglitazone prevents ferroptosis and lessens MASH brought on by arsenic. Nicorandil may prevent ferroptosis and the translocation of ACSL4 into the mitochondria. By preventing ACSL4 activity, triacsin C can alleviate parkinson’s disease (PD); and clausenamide can also alleviate behavioural impairments in PD animal models by preventing the nuclear translocation of ALOX5. Methyl ferulic acid controls the expression of ACSL4 to reduce neuropathic pain in mice. By reducing the expression of ACSL4 and ALOX15 in spinal cord tissue, proanthocyanidin therapy dramatically improves spinal cord damage (Wei et al., 2020; Guo et al., 2023; Li et al., 2023f; Chen et al., 2024; Huang et al., 2024; Duan et al., 2022b; Iqbal et al., 2023; Tang et al., 2023; Li et al., 2023g; Liu et al., 2023b; Zhou et al., 2020b; Cheng et al., 2020). To summarize the potentiali therapeutic agents against ferroptosis in various neurodegenerativ and neuropsychatric diseases, we provided a representative table below.

## Table of potential therapeutic candidtes

**Table:** 

Drugs	Model	Dose	Mechanism	Signal	Refs
Salidroside	Aβ1−42-induced AD model glutamate-induced HT-22 cell AD model	*In vivo*: 50 mg/kg *In vitro*: 10, 20,40, 80, 160, 320 μM	Nrf2/HO-1 pathway ↑	SOD, GSH, GPX4, SLC7A11 ↑, ROS, Fe2+, MDA ↓	Yang et al. (2022)
Edaravone	C57BL/6J mouse CSDS model	10 mg/kg	RTA	GSH, SOD, GPX4, GSH-PX, Nrf2, HO-1 ↑ MDA, ROS ↓	Dang et al. (2022)
Phenothiazine derivative 51	MCAO-induced SD rat stroke model	0.01, 0.1, 1 μM	RTA	GSH ↑ ROS, MDA ↓	Yang et al. (2021)
Resveratrol	MCAO-induced SD rat stroke model OGD/R-induced primary cortical neuron stroke model	*In vivo*: 30 mg/kg *In vitro*: 5, 10, 20 µM	RTA	GPX4, GSH ↑ ROS, ACSL4, Fe2+ ↓	Zhu et al. (2022)
Vitamin E	PTZ-induced SD rat chronic epilepsy model	200 mg/kg	ALOX inhibitor	GPX4, GSH ↑MDA, ROS, 15-LOX ↓	Zhang et al. (2022c)
Baicalein	FeCl3-induced C57BL/6J mouse PTE model FAC-induced HT-22 cell PTE model	*In vivo*: 100 mg/kg, *In vitro*: 1, 2, 4, 8, 16, 32 μM	ALOX inhibitor	GPX4 ↑ ROS, PTGS2, 4-HNE, 12/15-LOX ↓	Li et al. (2019)
Zileuton	Glutamate-induced HT-22 cells	1, 10, 50, 100 µM	ALOX inhibitor	ROS, 5-LOX, lipid peroxidation ↓	Liu et al. (2015)
Vilda	Collagenase-induced C57BL/6J mouse ICH model	50 mg/kg/d	DPP-4 inhibitor	GPX4 ↑ MDA, Fe2+ ↓	Zhang et al. (2022d)
GKT137831	PQ- and maneb-induced SHSY5Y cells	0.5 μM	NOX inhibitor	GSH, GPX4 ↑ ROS, MDA ↓	Hou et al. (2019)
Baf-A1	6-OHDA-induced PC12 cell PD model	100 nM	Autophagy inhibitor	GPX4, FTH1 ↑ NCOA4 ↓	Tian et al. (2020)
CPX	Glutamate-induced OHSC	5 μM	Iron chelator	ROS ↓	Dixon et al. (2012)
DFO	FAC-induced PC12-NGF cell PD model	Unknown	Iron chelator	GPX4, FTH1 ↑ DMT1, TfR1, FPN, ACSL4, ROS ↓	Zen et al. (2021)
Lip-1	RSL3-induced OLN-93 cell line SCI model	1 μM	RTA	GPX4, GSH, FSP1 ↑ MDA, ROS ↓	Fan et al. (2021)
Fer-1	Collagenase-induced C57BL/6 mouse ICH model Hb-induced OHSC ICH model	*In vivo*: 1 pmol of Fer-1 *In vitro*: 10 μM	RTA	MDA,4-HNE, ROS, PTGS2 ↓	Li et al. (2017)

One popular technique for enhancing the bioavailability and retention duration of bioactive substances is the use of nanoparticles. Quercetin’s bioavailability is a significant concern and a major barrier to its application in AD treatment. Liu et al. created a smart nanoparticle (TQCN) to treat AD by addressing ferroptosis. It was made from quercetin and modified with triphenylphosphine. TQCN, a specific type of nanomedicine efficiently chelates iron by spontaneous coordination mediated by plant polyphenols and self-assemble metal-phenol nanocomplexes *in situ*, reducing iron overload and related free radical outburst by utilizing advantageous brain targeting and mitochondrial localization features. TQCN also lowers cellular lipid peroxidation, restores iron metabolism balance, and activates the Nrf2 endogenous defence system. Due to its multimodal modulation of the pathogenic process that causes ferroptosis, TQCN therapy may alleviate severe cognitive impairment in AD mice and relieve a variety of neurodegenerative illnesses associated with brain iron buildup (Liu et al., 2024b; Herpich and Rincon, 2020). Neurotrophin, nerve growth factor, and edaravone are examples of neuroprotective medications that protect the brain from ferroptosis and oxidative stress. However, because of their short circulation half-life and limited BBB permeability, these neuroprotective medications frequently fall short of the anticipated therapeutic effect. Zhang et al. used the acidic pathological features of ischaemic tissue to build a pH/GSH-supported polyamino acid nanogel (NG/EDA). To increase the neuroprotective effects of edaravone, NG/EDA is triggered by the acidic and edaravone-induced high levels of GSH microenvironment. This allows for the selective and prolonged release of edaravone at the site of ischaemic injury. The findings demonstrated that in rats with pMCAO, NG/EDA could effectively accumulate at the site of cerebral ischaemia damage and cross the blood-brain barrier. By preventing ferroptosis, NG/EDA dramatically increases the survival rate of OGD neurones while also considerably lowering the infarct volume and neurobehavioral score of pMCAO mice. A novel and promising model for neuroprotection in cerebral I/R injury and other illnesses of the central nervous system may be offered by this pH/GSH dual-responsive nanoplatform. Inflammatory cytokine production has a key role in the pathophysiology of disorders involving I/R damage. A class of copper-based, neutrophil membrane-coated nanoparticles (N-Cu5.4O@DFO NPs) with excellent stability and biocompatibility was described by Ding et al. By efficiently scavenging iron and exhibiting strong antioxidant qualities, these nanoparticles reduce oxidative damage and inflammatory reactions, thereby enhancing I/R damage (Herpich and Rincon, 2020; Jin et al., 2017; Zhang et al., 2024c; Zhuge et al., 2024; Ding et al., 2023). A flavonoid glycoside obtained from locust plants, rutin has strong antioxidant properties and has been widely used to treat neurological and cardiovascular conditions. To get rid of ROS and stop ferroptosis, Feng et al., created rutin-loaded polydopamine nanoparticles (PEG-PDA@rutin NPs). PEG-PDA@rutin NPs have a diameter of roughly 100 nm and demonstrate both ROS-triggered drug release and superior ROS clearance capabilities. PEG-PDA@rutin NPs have the ability to efficiently enter cells, stop ferroptosis, remove ROS, and heal mitochondrial damage. Ren et al. developed a ROS-responsive drug nanocore, mPEG-b-Lys-BECI-TCO, for SCI repair, and combined MSCs with Fer-1 to create a synergistic drug release nanoparticle system (Niu et al., 2021; Muvhulawa et al., 2022; Negahdari et al., 2021; Feng et al., 2024b; Zhang et al., 2021). Following SCI, this multimodal therapy approach may prevent inflammation and ferroptosis and provide a fresh approach to building drug-synergistic cell treatment systems that target ferroptosis. An innovative flavonoid glycoside with potent antioxidant properties is apigenin-7-O-glucoside (AGL). By selectively binding to HO-1 and monoamine oxidase b, AGL helps to avoid ferroptosis and preserve mitochondrial function by preventing the buildup of Fe2+ and the generation of ROS. However, AGL’s limited practical use is due to its weak water solubility. Zhao et al. created two amphiphilic compounds, mPEG-TK-DA and DTPA-N10-10, with ROS-scavenging properties in order to get around this restriction. They also self-assembled AGL through hydrophobic and hydrophilic contacts, creating multi-site ROS-scavenging nanoparticles known as PDN@AGL. By lowering ROS levels and lipid peroxidation, PDN@AGL prevents ferroptosis, and it is thought that the ATF3/SLC7A11 pathway is a key player in this process. The possible use of PDN@AGL to treat human disorders is supported by the control of ATF3/SLC7A11-mediated ferroptosis. PDN@AGL offers a promising treatment approach for conditions marked by ferroptosis and oxidative stress by resolving the solubility problem and boosting AGL’s antioxidant capability (Yao et al., 2023; Feng et al., 2022; Zhao et al., 2024b; Katz et al., 2021).

## CRISPR/CAS9 based therapeutic strategies

**FIGURE 6:**
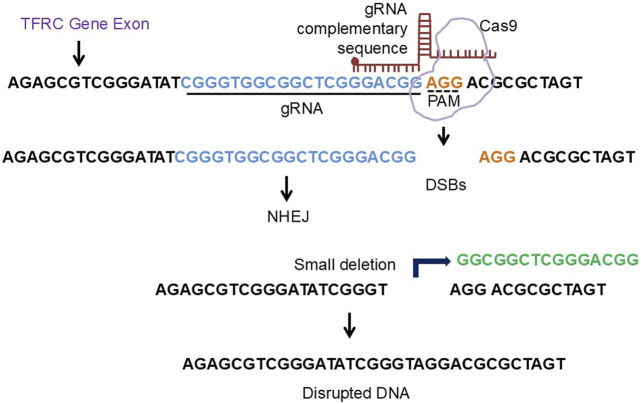
The diagrammatic representation of crispr/cas9 approach to engineer ferroptosis related gene. The TFRC gene exon FASTA sequence was copied from NCBI and the respective gRNA were designed using CHOP CHOP software. The deletion of the nucleotide sequence from the corresponding exon will lead to knockdown of the TFRC gene.

A novel therapeutic approach for both central and peripheral disorders is gene level editing. Gene editing techniques like CRISPR/CAS9 can be used to treat diseases caused by genetic mutations, according to a number of well-known studies. For example, Transferrin Receptor Protein 1 gene, after downloading the FASTA sequence (mRNA) from NCBI and copying the exon (the coding sequence), sgRNA was created to the crosponding exon. In the present review, we provide a research direction that by deleting a tiny amount of DNA from the relevant exon, the gene of interest will be knocked down, thereby preventing ferrosptsis-mediated degeneration (Konstantinidis et al., 2022; Wadhwani et al., 2019; Kolanu, 2024; Abdelnour et al., 2021; Deneault, 2024) (Figure 6).

## Challenges and future directions

Despite the promise of ferroptosis inhibition in neuroprotection, several challenges remain. One major obstacle is the complexity of ferroptosis regulation in the brain and its intricate interplay with other cell death pathways, including apoptosis, autophagy, and necroptosis. Dissecting the precise molecular mechanisms underlying ferroptosis and its pathological role in neurodegeneration remains a significant research priority.

Iron dysregulation is central to ferroptosis, and therapies targeting iron homeostasis, such as chelation, have shown mixed clinical success. Iron chelators like deferoxamine and deferiprone reduce labile iron and mitigate reactive oxygen species (ROS), yet they face limitations in crossing the BBB effectively. Emerging strategies, such as nanoparticle-based delivery systems, could improve BBB penetration, allowing targeted chelation therapies to reach affected brain regions (Nunez and Chana-Cuevas, 2018; Popescu and Nichol, 2011). Additionally, mapping brain metals using advanced imaging techniques, such as quantitative susceptibility mapping (QSM), offers a promising non-invasive approach to identify iron deposition and monitor therapeutic responses in diseases like AD (Uchida et al., 2022). However, challenges remain in standardizing QSM and interpreting regional brain iron concentrations across diverse neurodegenerative disorders (Ward et al., 2014). Genetic factors, such as PRMT1 expression, also complicate ferroptosis regulation. PRMT1 promotes ferroptosis by suppressing key antioxidant systems, including solute carrier family 7-member 11 (SLC7A11), and its inhibition could provide dual therapeutic benefits—enhancing neuroprotection while improving treatment responses in conditions like gliomas (Li et al., 2024d). Advanced genome-editing approaches, particularly CRISPR/Cas9-based techniques, hold significant promise for precisely targeting ferroptosis-related genes to attenuate neurodegeneration (Nouri Nojadeh et al., 2023). However, ensuring the safety and specificity of CRISPR-based interventions in the central nervous system (CNS) remains a challenge. Delivery systems such as adeno-associated viruses (AAVs) offer a potential solution for CNS-specific targeting of ferroptosis regulators but require further optimization and validation in preclinical models. Another challenge is the intersection of ferroptosis with neuroinflammatory pathways. Excess iron accumulation in the substantia nigra, as seen in PD, exacerbates oxidative stress and neuroinflammation, further driving dopaminergic neuronal loss. Neuroinflammatory cytokines and immune activation pathways, such as NF-κB signaling, may synergize with ferroptosis to amplify neurodegeneration. Addressing both iron dysregulation and inflammatory processes will require combination therapies that target multiple pathological pathways simultaneously.

In amyotrophic lateral sclerosis (ALS), elevated ferritin and transferrin receptor levels in cerebrospinal fluid have been associated with reduced survival, suggesting that disrupted iron metabolism may serve as both a biomarker and a therapeutic target. However, identifying patient-specific factors, such as genetic predispositions or iron regulatory gene polymorphisms, will be crucial for tailoring ferroptosis inhibitors to individual patients. Biomarkers like serum ferritin, oxidative stress markers, and QSM-based iron mapping may aid in predicting therapeutic responses and monitoring disease progression. Lastly, syndromes of neurodegeneration with brain iron accumulation (NBIA), such as pantothenate kinase-associated neurodegeneration (PKAN), exemplify the devastating clinical effects of ferroptosis driven by excessive iron deposition. NBIA disorders often present with progressive dystonia, parkinsonism, and cognitive decline, highlighting the urgent need for therapies that prevent ferroptosis-mediated neuronal loss (Ward et al., 2014; Ayton et al., 2017; Nadjar et al., 2012; Belaidi and Bush, 2016; Schneider and Bhatia, 2012). Overcoming these challenges requires a multifaceted approach involving novel drug delivery systems, advanced imaging modalities, and genetic targeting technologies to optimize therapeutic efficacy and safety.

## Conclusion

Ferroptosis represents a novel and distinct form of regulated cell death, characterized by iron dysregulation, lipid peroxidation, and oxidative stress. Its involvement in neurodegeneration with brain iron accumulation highlights its pathological significance. The evidence linking ferroptosis to neuronal loss underscores its potential as a critical driver of neurodegeneration in regions of the brain where iron accumulation and oxidative damage are pronounced (Ayton et al., 2017; Belaidi and Bush, 2016; Schneider and Bhatia, 2012). A sophisticated understanding of the molecular mechanisms regulating ferroptosis—including disruptions in the antioxidant defense system, iron homeostasis, and lipid metabolism has paved the way for identifying therapeutic targets to mitigate neurodegeneration and preserve neuronal function.

Current therapeutic approaches targeting ferroptosis hold promise for neuroprotection. Strategies such as iron chelation therapy, antioxidant supplementation, and small-molecule ferroptosis inhibitors have shown efficacy in preclinical models by reducing oxidative stress, limiting lipid peroxidation, and restoring iron homeostasis. Advanced therapies, such as CRISPR/Cas9-based gene editing, offer a precise means to target ferroptosis-related genes (Li et al., 2024d; Zhang et al., 2024d). Furthermore, innovations in nanoparticle-based drug delivery systems and brain-penetrant chelators address longstanding challenges related to therapeutic access across the blood-brain barrier. These advancements suggest that a combination of pharmacological, genetic, and nanotechnological approaches may offer synergistic benefits for slowing or halting disease progression (Ashok et al., 2022; Zhou et al., 2024).

Despite these promising developments, significant challenges remain in translating ferroptosis-targeting therapies to clinical practice. The complex interplay between ferroptosis and other cell death pathways, such as apoptosis, autophagy, and neuroinflammation, necessitates further investigation. Identifying robust biomarkers, such as serum ferritin levels, oxidative stress markers, and advanced neuroimaging techniques like quantitative susceptibility mapping (QSM), will be necessary for patient stratification and therapeutic monitoring (Ward et al., 2014; Ayton et al., 2017; Nadjar et al., 2012). Additionally, understanding the heterogeneity of ferroptosis mechanisms across different neurodegenerative diseases and patient populations will enable the development of tailored therapies.

In conclusion, targeting ferroptosis provides a promising therapeutic avenue for combating neurodegenerative diseases marked by iron dysregulation and oxidative damage. Continued research into the molecular mechanisms of ferroptosis, coupled with advancements in therapeutic delivery and biomarker development, is essential for realizing its clinical potential. By integrating multidisciplinary approaches and addressing current challenges, ferroptosis-targeted strategies hold the potential to transform the treatment landscape for neurodegenerative disorders, ultimately improving outcomes and quality of life for affected individuals.
