# Supplementary figures and images for: Targeting ferroptosis for neuroprotection: potential therapeutic avenues in neurodegenerative and neuropsychiatric diseases
Source: Front Physiol. 2025 Aug 28;16:1641323. doi: 10.3389/fphys.2025.1641323 (PMC12423103; doi:10.3389/fphys.2025.1641323)

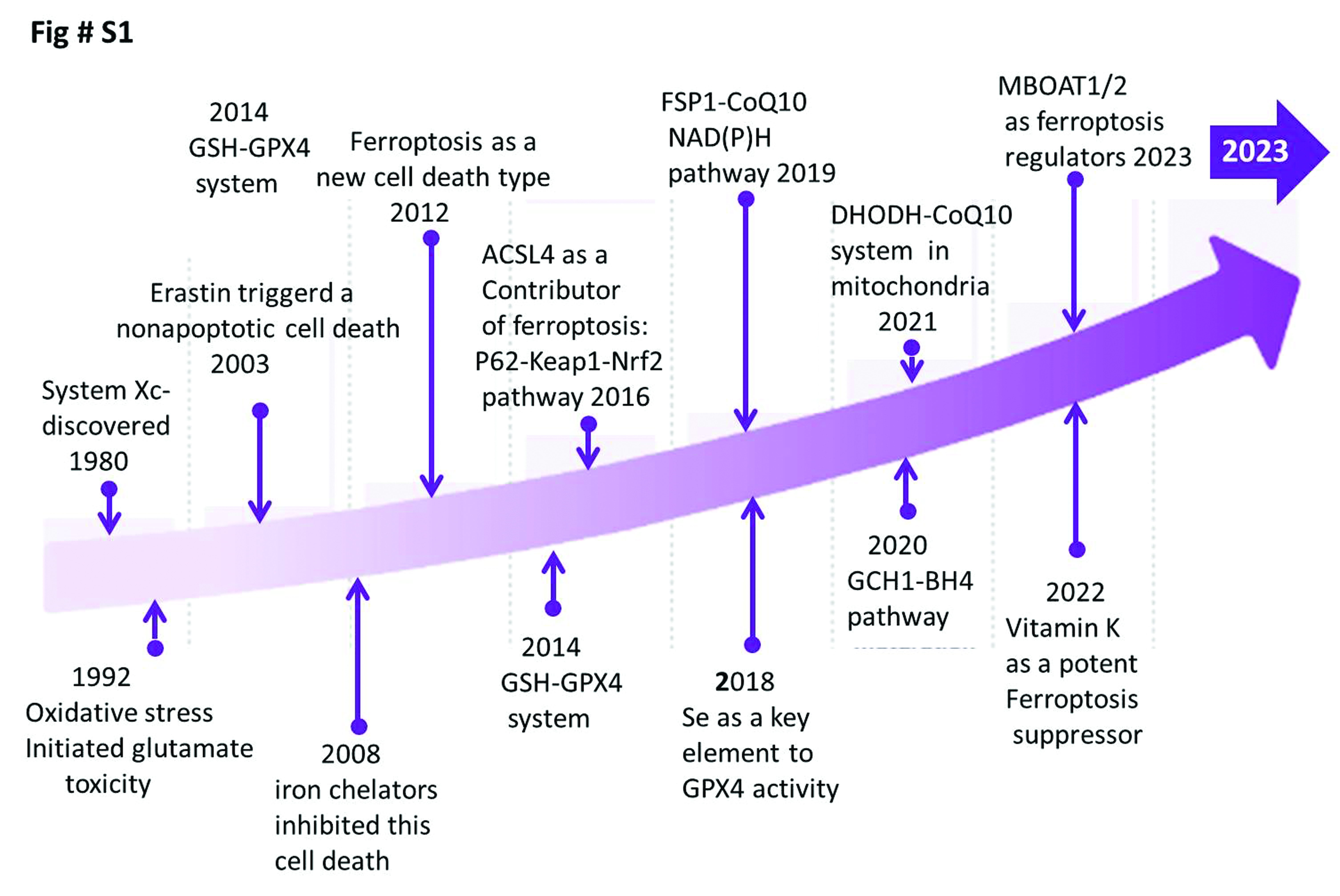

Supplement: Supplementary file 1 [file Image1.jpeg]
